# Dental Practice Websites in Germany—How Do They Inform about Fluoridation?

**DOI:** 10.3390/dj12030065

**Published:** 2024-03-04

**Authors:** Antje Geiken, Mirja Kock, Lisa Banz, Falk Schwendicke, Christian Graetz

**Affiliations:** 1 Clinic of Conservative Dentistry and Periodontology, University of Kiel, 24105 Kiel, Germany; mirja.kock@googlemail.com (M.K.); lisa.banz@gmx.de (L.B.); graetz@konspar.uni-kiel.de (C.G.); 2 Clinic for Conservative Dentistry and Periodontology, University Hospital of Ludwig-Maximilians-University Munich, 80336 Munich, Germany; falk.schwendicke@med.uni-muenchen.de

**Keywords:** fluoride, prevention, internet, dental website, medical information

## Abstract

Fluoridation (Fl) is effective in preventing caries; however, it is unclear to what extent its use is counteracted by misinformation on the internet. This study aimed to evaluate the information provided on professional websites of German dental practices regarding fluoridation. A systematic search was performed by two independent examiners, utilizing three search engines, from 10 September 2021 to 11 December 2021. Modified, validated questionnaires (LIDA, DISCERN) were used to evaluate technical and functional aspects, generic quality, and risk of bias. Demographic information and statements about Fl were also collected. The intra- and inter-rater reliability assessments were excellent. Of the 81 websites analyzed, 64 (79%) mentioned Fl, and 31 (38%) indicated it as a primary focus. Most websites met at least 50% of the LIDA (90%) and DISCERN criteria (99%), indicating that the general quality was good. Thirty (37%) of the websites explained the impact of Fl, and forty-five (56%) indicated an opinion (for/against) on Fl. The practice location and the clinical focus were not associated with the overall quality of websites. Only a minority of websites explained the effects of Fl. Taken together, this study highlights that there is a distinct lack of good-quality information on FL.

## 1. Introduction

Fluoridation is a crucial component of modern caries-preventive dentistry and takes the form of regular mechanical plaque removal at home, as well as potential supplementary professional teeth cleaning in a dental practice. The relationship between fluorides and a reduction in caries prevalence is well established [1].

Fluorides are predominantly recommended for local application, for example, at home using fluoride-containing toothpaste or in the form of highly concentrated varnishes or gels at a dental practice. Used in appropriate dosages, fluoride is safe [2] and has been linked with a global decline in caries incidence in childhood and adolescence. As a result, international bodies like the World Health Organization (WHO) recommend fluoride application [3], and many countries have also formulated recommendations to maximize the benefits of fluoride use [4].

However, a large proportion of the population may obtain health-related information not via such sources [5] but rather through the internet, where the quality of the information varies considerably [6,7]. Conversely, it can be observed that the role of artificial intelligence [8], social media [9,10], and manipulated health information is increasingly scrutinized [10].

In dentistry, fluoridation can potentially be seen as controversial [11,12]. In too high a dosage, fluorides may have negative effects on general health [13], with misinformation about the safety of fluorides being widely present on the internet [14].

Inadequately informed patients can strain the doctor–patient relationship [15,16], emphasizing the importance of providing them with scientifically backed information. 

Dentists, as experts in their field, have a role in providing accurate information on their own websites, reflecting the latest state of science and not unsubstantiated personal opinions. Previous studies dealing with a systematic analysis of dental websites have focused on topics such as periodontitis or restorative care and have so far painted an unsatisfactory picture of the quality of information provided [17,18]. Currently, no analysis of information from German dental practices websites on fluoride and fluoridation has been conducted. 

Such studies must always additionally consider aspects such as whether it is technically possible to provide the patient with information via the website (e.g., can the websites be opened without prior registration) and the information’s author. To aid in this aspect, criteria catalogs can be utilized, and one catalog, called LIDA, was developed to provide three levels of data (accessibility, usability, and reliability) with sub-categories for a standardized analysis [19]. Additionally, the DISCERN criteria catalog was developed to check the general provision of information (e.g., the origin of website information) and to evaluate a website’s information in terms of its reliability and quality [20,21].

The aim of this study was to evaluate the information provided on fluoride and dental fluoridation by the websites of German dental practices in terms of technical and functional aspects (LIDA criteria), as well as the overall quality and risk of bias (DISCERN criteria).

## 2. Materials and Methods

### 2.1. Sample Size Calculation

Sample size estimation was adopted from a previous study in this domain [18]. A significance level of α = 0.05, an effect size of 0.2 (moderate), and a power of 1 − β = 0.80 were used to calculate the required number of websites. The estimate indicated that at least 64 sites should be considered sufficient (F = 2.52; df = 4, expected power 81%).

### 2.2. Functional Aspect and Generic Risk of Bias

A data extraction form was used to collect content-related items, i.e., demographic data (size of the city, structure of the practice, age of the dentists, specialization) (Table 1). The established LIDA and DISCERN criteria catalogs were used to analyze the data [17,18,22,23,24].

LIDA captures criteria such as the accessibility, usability (including clarity, consistency, functionality, and engagement), and reliability (timeliness, conflicts of interest, and content creation) of websites. LIDA analyzes the clarity of the website, the function of all navigation links, the use of third-party plug-ins (e.g., PDFs), interactivity, topicality, and the existence of a search or comment function, as well as the financing of the website and who is responsible for hosting it. Items are weighted on an ordinal scale of 0 (never), 1 (occasionally), or 2 (always) (Table 2).

DISCERN (quality criteria for health information for consumers) assesses the user experience, the overall quality and risk of bias of health information for treatment decisions, the reliability (trust in information and information source), and the quality of information (treatment alternatives). As with LIDA, DISCERN uses an ordinal scale of 0 (never), 1 (occasionally), or 2 (always) (Table 3). This means that DISCERN is used to check the page information in general but not topic-specific factors.

In both the LIDA and DISERN criteria catalogs, the points achieved are added up to a total score, and then the percentage of the maximum possible score is calculated. A score of >90% stands for good results; a score of <50% stands for poor results.

Furthermore, fluoride-specific information was collected covering fluoride use (general information on fluoride, recommendations, and content aspects of fluoride), forms of therapy (fluoridation), and consumer advice on fluoride sources (Table 4). The information was rated on a Likert scale, where 0 stands for no information and 2 for complete information. This criteria catalog was designed by five dentists (three specialists in conservative dentistry and two specialists in pediatric dentistry).

### 2.3. Search Strategy

A systematic search using the German search terms “Dentist/Fluoridation/Fluoride”, separated by slashes, was performed by two independent investigators (M.K., L.B.) in Germany between 10 September 2021 and 11 December 2021, using three search engines (Google, Bing, Yahoo). Prior to this, browsing history and cookies were deleted. Only websites from German-language dental practices (based in Germany) were included; websites from university clinics, health insurance companies, health services, and blogs were excluded. 

**Figure 1:**
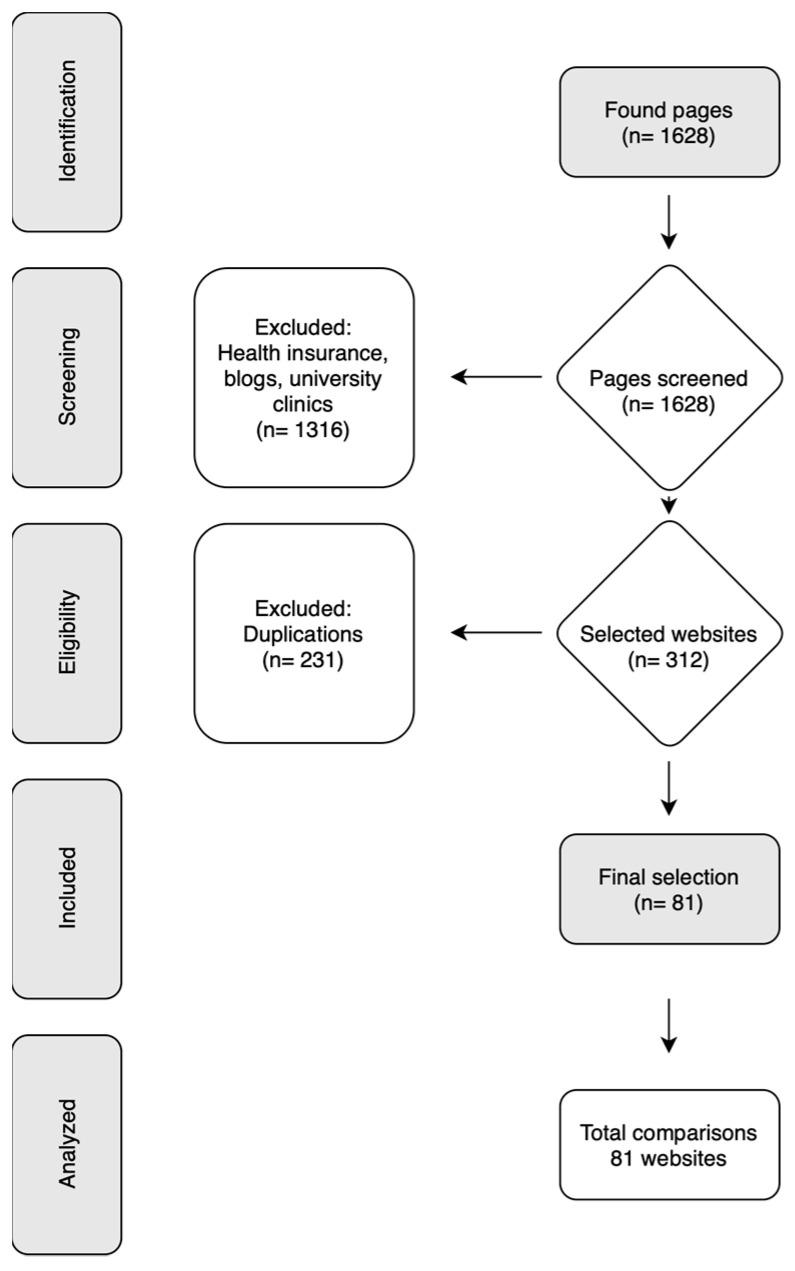
Flowchart of search and selection results.

In all, 1628 websites were found and filtered. A total of 1316 were websites from university hospitals, health insurance companies, or blogs and were excluded. A further 231 websites were removed as they were duplicated. The final selection analyzed consisted of 81 websites from German dental practices (Figure 1).

For quality assurance, the intra-rater and inter-rater reliability were collected; for this purpose, ten websites were evaluated consecutively. Re-evaluation occurred at 3-month intervals (first evaluation 9 October 2021 to 9 December 2021, second evaluation 11 December 2021). The raters discussed possible discrepancies, and a third rater’s (A.G.) assessment was obtained if necessary.

### 2.4. Data Extraction and Analysis

The two reviewers independently transferred data from the included websites to a data extraction form. The following data were extracted from the websites: demographic information (practice name, URL, practice location [rural ≤ 5000, city > 5000–<100,000 inhabitants, large city ≥ 100,000 population], practice setting (single practice, multi-practice, or chain practice), age of the dentist(s) (young: younger than 41 years, middle-aged: 41 to 50 years, older: older than 50 years; determined from CVs on the websites, averaged for multi-practice or chain practices), specialization (no specification, any specialization, pediatric dentistry)), and specific fluoridation information (0 = no specification, 2 = complete specification) (Table 1, Table 2, Table 3 and Table 4).

### 2.5. Statistical Analysis

Statistical analysis was performed using SPSS Statistics for Mac 28.0.0.0 (IBM, Chicago, IL, USA). Statistical significance was set at *p* < 0.05. Data were primarily analyzed descriptively. Medians, quartiles, ranges, and a quality score (relative percentage: website score for all corresponding items divided by the maximum possible total score) were calculated for each domain and the total domains. Besides the Chi-square coefficient, generalized linear modeling was used to assess the relationship between practice-related characteristics and overall quality (in %). Interaction terms were not used in this analysis, as this would require additional model development and risk alpha inflation. 

## 3. Results

A total of 81 websites were analyzed (Figure 1), and a high inter-/intra-rater reliability indicated a robust analysis methodology (>0.9). The websites were mainly maintained by dentists of middle age (41–50 years; 40%) or older age (>50 years; 35%). Only 11% were aged 40 years or less, and 15% of websites did not provide information on the age of the dentist(s). Practices were mostly situated in cities (50%) or large towns (41%), with the majority (68%) of dentists working in multi-treatment practices. The number of chain practices (2%) was negligible, and the proportion of single-treatment practices was 30%. The specializations of the dentists were predominantly not reported (77%) (Table 1). 

The evaluation of the DISCERN criteria showed that for 80 websites (99%), at least 50% of the criteria were met. The review of the LIDA criteria showed that at least 50% of the criteria were met by 73 websites (90%). All websites were free of charge and could be used without registration or subscription. The design, structure, and navigation were comprehensible and technically usable; however, most of the websites did not provide a search or comment function. It was evident who had created the page and which sources of information had been used for it, and all the pages examined were updated regularly (Table 2). In most cases, it was evident when the website was created and whether it contained detailed information about supplementary aids and information (Table 3). In only a few instances, it was recognizable whether the web page was written independently or under the influence of advertising. 

Information was provided about the existence of different fluoridation procedures, their mode of action, and their benefits. The consequences of not using fluorides and the influence of fluorides on quality of life were mentioned rarely. Further analysis revealed that in 64 websites (79%), the term fluoride was mentioned, and in 31 (38%), it was indicated as a focus. Thirty websites (37%) explained fluoride’s effects, 21 (26%) addressed the risks of non-use, and 45 (56%) referred to their point of view on fluoride use (for/against). A distinction between basic and intensive prophylaxis was not found in the websites analyzed. Moreover, there was no mention of age factors or intake instructions, such as dosage, advantages of early administration, or disadvantages of non-administration (Table 4). In the multivariable analysis, most practice-related factors were not significantly associated with the overall quality of the website (Table 5). However, chain practices were associated with a slightly higher quality (0.8; 95% CI of −1.7 to 3.4). 

## 4. Discussion

Fluorides are one of the most important pillars of caries prevention [4] and an essential part of early childhood prophylactic dentistry. Nevertheless, their preventive use in dentistry remains controversial. The arguments put forward against fluoridation are often based on incomprehensible evidence that is not scientifically proven [12,25]. This can lead to misunderstandings, as interested parents or patients themselves are increasingly looking for background knowledge, and currently, more and more information is being obtained via the internet (e.g., social media such as Facebook, Twitter, and YouTube) and not directly from the dentist [26]. Indisputably, social media has made the provision of medical information very easy for patients. Importantly, these data are not checked for their scientific accuracy, which means that false information can be easily spread [27,28,29]. As mentioned by Wang and colleagues, we have to accept this “era of fake news” [26], and the fact that patients are unable to distinguish between scientifically sound and false information means that there is a very high risk of misinformation. Often, it is difficult for parents to distinguish between true and false information in the medical domain. Receiving contradictory information about fluoridation in children can therefore quickly lead to misjudgments about the correct use of fluorides [30]. A recently published study showed that parents have difficulties with managing their children’s oral health without guidance [31]. This covered choosing the right toothpaste for their child’s age and determining the correct amount of toothpaste to use, which has a direct impact on the use of fluoride in children [31]. By providing specialist articles on their websites, dentists have a major opportunity to influence the correct provision of information for patients. 

To the best of our knowledge, no studies have been carried out on the quality of fluoride information on dental websites for German dental practices. Thus, the evidence on the quality of dental websites is limited. However, selective data are available on orthodontic and periodontal treatments, restorative dentistry, and craniomandibular dysfunction [17,18,23,32]. LIDA and DISCERN were used in these studies to assess the quality and functionality of the websites [17,18,23,29,32] and can therefore be seen as a gold standard for assessing the quality of technical aspects, overall quality, and risk of bias. In the current study, DISCERN and LIDA received high scores with at least 50% of the criteria met in 90–99% of all websites, which means that the websites examined were of good quality. Moreover, similar studies on German dental websites displayed comparable results [17,18]. Unfortunately, the quality of the fluoridation information painted the opposite picture. 

The quality of these data was inadequate. The term fluoridation was only mentioned on 64 websites (79%), while it was only mentioned as a focus on 31 websites (38%). A definition of the term “fluoride” was given on 34 websites (42%), and “fluoridation” was defined on 23 (28%). Most worrying was the distinct lack of information on the effects of fluoride, with only 30 websites (37%) covering this information. There are myths circulating on the internet about the effects of fluoride, which is why the dental profession must be particularly careful when providing information [14,16,29]. This is particularly important as misunderstood information about fluoridation can be passed on by other medical professionals such as midwives or pediatricians, leading to further confusion for patients and parents [33,34]. 

As mentioned by previous studies, no significant correlation was found between fluoridation and the dental practice location or setting (single practice, multi-practice, or chain practice) or the age of the dentist on the investigated websites [17,18]. However, the websites examined in the earlier studies showed better quality in the communication of dental content on periodontitis by specialized dentists [18]. Unfortunately, due to the very low proportion of German dentists specializing in pediatric dentistry, our study was probably not conclusive enough for such an analysis. 

The present study has limitations and must be interpreted with this in mind. The websites were analyzed in a national context, i.e., only websites from Germany and in German were included. Due to national factors, e.g., specialization in the context of specialist training, the results are not internationally comparable. Additionally, the German national common health care insurance system and requirements of regional dental associations will have influenced the website designs/contents, and national regulations and specifications regarding data protection have impacted the design of dental practice websites. 

However, the evaluation procedures used (LIDA and DISCERN) are considered well established, and the evaluation by two independent investigators (L.B. and M.K.) also showed very high values for intra- and inter-rater reliability. Unfortunately, there was previously no established evaluation catalog for assessing the quality of information on fluoridation. The assessment catalog used in this study was newly developed and validated together with a group of pediatric dentists and other specialist dentists.

## 5. Conclusions

Fluoridation is essential in dentistry, and as more people use the internet to inform themselves about medical issues, the results of this study are highly relevant. Fortunately, most dental websites in Germany mention fluoride; however, a deficit in the quality of information was revealed as only a few websites explained the effects of fluoridation and its possible advantages/disadvantages. Therefore, further efforts must be made by dental associations and organizations to improve the quality of information on the websites of dental practices. We must always remember that these websites are considered a trustworthy source of information by patients who use them.

## Figures and Tables

**Table 1:** Practice-specific parameters of the included websites.

Variable and Attribute	Value (n (%))
**Practice location**	
Rural	7 (9%)
City	41 (50%)
Large city	33 (41%)
**Practice setting**	
Single practice	24 (30%)
Multi-practice	55 (67%)
Chain practice	2 (3%)
**Age**	
Young	9 (11%)
Middle-aged	32 (39%)
Older	28 (35%)
No information	12 (15%)
**Specializations**	
Pediatric dentistry	3 (4%)
Any specializations	17 (17%)
No information	62 (79%)

**Table 2:** Domains regarding technical and functional aspects were assessed using the modified LIDA instrument (version 1.2). Scores of 0, 1, and 2 were used [18].

Item	Median (IQR ^a^; Min–Max ^b^)
**1.1 Accessibility**	
• Is the information available full text without registration, log-in or subscription?	2 (0; 2–2)
**1.2 Usability**	
• Is there a clear statement of who this website is for?	2 (0; 1–2)
• Is the level of detail appropriate to their level of knowledge? Is the layout of the main block of information clear and readable?	2 (0; 1–2)
• Is the navigation clear and well structured?	2 (0; 0–2)
• Can you always tell your current location on the site?	2 (0; 1–2)
• Do navigational links have a consistent function?	2 (0; 0–2)
• Does the site provide an effective search function?	2 (0; 0–2)
• Can you use the site without third party plugins?	0 (0; 0–2)
• Can the user make an effective judgment of whether the site applies to them?	1 (0; 0–2)
• Is the website interactive?	2 (0; 1–2)
• Does the website integrate non textual media?	1 (0; 0–2)
**1.3 Reliability**	
• Can users submit comments on specific content?	0 (0; 0–0)
• Is site content updated at an appropriate interval?	2 (0; 1–2)
• Is it clear who runs the site?	2 (0; 0–2)
• Is it clear who pays for the site?	1 (0; 1–2)
• Can the information be checked from original sources?	2 (0; 1–2)

^a^ IQR: interquartile range. ^b^ min–max: minimum and maximum. 0 = never, 1 = occasionally, 2 = always.

**Table 3:** Domains regarding generic quality and risk of bias were assessed using the modified DISCERN instrument. Scores of 0, 1 and 2 were used [18].

Item	Median (IQR ^a^; Min–Max ^b^)
**2.1 Reliability**	
• Are the aims clear?	2 (0; 1–2)
• Is it clear what sources of information were used to compile the publication?	0 (0; 0–2)
• Is it clear when the information used or reported in the publication was produced?	2 (0; 0–2)
• Is it balanced and unbiased?	1 (0; 0–2)
• Does it provide details of additional sources of support and information?	1 (0; 0–2)
• Does it refer to areas of uncertainty?	0 (0; 0–1)
**2.2 Quality**	
• Does it describe how each treatment works?	2 (0; 0–2)
• Does it describe the benefits of each treatment?	2 (0; 0–2)
• Does it describe the risks of each treatment?	0 (0; 0–2)
• Does it describe what would happen if no treatment is used?	1 (0; 0–2)
• Does it describe how the treatment choices affect overall quality of life?	1 (0; 0–2)
• Is it clear that there may be more than one possible treatment choice?	2 (0; 0–2)
• Does it provide support for shared decision making?	1 (0; 0–2)

^a^ IQR: interquartile range. ^b^ min–max: minimum and maximum. 0 = never, 1 = occasionally, 2 = always.

**Table 4:** Fluoridation-specific aspects. Scores between 0 and 2 were used.

Item	Median (IQR ^a^; Min–Max ^b^)
• Is the word fluoridation mentioned at all?	2 (0; 0–2)
• Is there an extra item that gives information only on fluoridation?	0 (0; 0–2)
• Is there a pro/con point of view?	2 (0; 0–2)
• Is fluoride explained in complete sentences?	0 (0; 0–2)
• Is fluoridation defined in complete sentences?	0 (0; 0–2)
• Is syst. and loc. fluoridation explained in more detail?	0 (0; 0–2)
• Is the mode of action explained in more detail?	0 (0; 0–2)
• Are the risks of non-administration explained?	0 (0; 0–2)
• Are the risks of excessive administration explained?	0 (0; 0–2)
• Is it divided into basic and intensive prophylaxis?	0 (0; 0–2)
• Is basis prophylaxis mentioned in more detail?	0 (0; 0–0)
• Is intensive prophylaxis mentioned in more detail?	0 (0; 0–0)
• Is information provided about the age at which fluoride should be taken?	0 (0; 0–2)
• Are fluoride sources mentioned?	0 (0;0–2)
• Are instructions for taking fluoride given?	0 (0; 0–2)
• Is information provided on the correct amount to take?	0 (0; 0–2)
• Are the advantages of early administration mentioned?	0 (0; 0–2)
• Are the disadvantages of not taking fluoride mentioned?	0 (0; 0–2)

^a^ IQR: interquartile range. ^b^ min–max: minimum and maximum. 0 = no information, 2 = complete information.

**Table 5:** Association between practice-related factors and the overall quality score. Significant associations are highlighted in bold.

Factor	Beta (Mean Quality Score)	95% Confidence Interval	*p*-Value
**Constant**	**15.3**	**12.8 to 18.0**	**<0.001**
Chained practice (ref.: multi-practice)	0.8	−1.7 to 3.4	0.52
Single practice (ref.: multi-practice)	0.1	−0.8 to 1.04	0.8
No information on specialization (ref.: pediatric dentistry)	−0.3	−2.8 to 2.3	0.84
Any specialization (ref.: pediatric dentistry)	0.2	−2.4 to 2.72	0.89
Rural (ref.: big city)	−0.34	−1.7 to 1.1	0.64
City (ref.: big city)	0.022	−0.8 to 0.8	0.96
Age of dentists (cont., per year)	0.4	−0.04 to 0.75	0.08
Number of dentists (cont.)	0.035	1.98 to 3.7	0.72

rural (≤5000), city (>5000–<100,000), large city (≥100,000).

## Data Availability

Data are contained within the article.
